# Audiologic and electrophysiologic evaluation in children with psychiatric disorders

**DOI:** 10.1016/S1808-8694(15)30844-2

**Published:** 2015-10-18

**Authors:** Carla Gentile Matas, Isabela Crivellaro Gonçalves, Fernanda Cristina Leite Magliaro

**Affiliations:** 1 PhD. Professor of Speech and Hearing Therapy - University of São Paulo Medical School.; 2 Speech and Hearing Therapy, MSc student - Graduate program in rehabilitation sciences - Human Communications - Department of Physical Therapy, Speech and Hearing Therapy and Occupation Therapy - University of São Paulo Medical School.; 3 MSc in Sciences - Graduate Program in Experimental Physiopathology - University of São Paulo Medical School. PhD student - Rehabilitation Sciences - Human Communication - Department of Physical Therapy, Speech and Hearing Therapy and Occupational Therapy - University of São Paulo Medical School. Speech and Hearing Program - Department of Physical Therapy, Speech and Hearing Therapy and Occupational therapy - University of São Paulo Medical School.

**Keywords:** auditory, evoked potentials, autism, hearing tests, asperger syndrome

## Abstract

Individuals with psychiatric disorders can present perception, attention and memory deficits, raising doubts about peripheral and/or central hearing loss. Thus, the aim of this study is to describe the audiological and electrophysiological results of individuals with psychiatric disorders, looking for peripheral and/or central auditory disorders. **Methods:** 20 individuals with autism and Asperger syndrome and 20 individuals without psychiatric disorders from eight to 19 years of age, were submitted to audiological and electrophysiological evaluation. **Results:** No alterations were observed on the audiological evaluation in all the individuals. In ABR, 50% of individuals with autism and 30% with Asperger syndrome presented alterations. Significant statistical differences were observed between the groups in the quantitative analysis. All groups presented alterations in AMLR and P300. In AMLR, no significant statistical differences were observed between the groups in the qualitative and quantitative analyses. In the P300, we noticed significant statistical differences between Asperger and control groups in the quantitative analysis. **Conclusions:** A high occurrence of alterations in auditory evoked potentials was seen in children with psychiatric disorders, although in some analysis it was observed a non-statistically significant difference when comparing study and control groups. We stress the need for a more careful investigation of the auditory function in this population.

## INTRODUCTION

Among psychiatric disorders in children we find the one so called Global Development Disorders, which represents a group of disorders characterized by qualitative alterations in reciprocal social interactions and communication modalities, and a set of restricted interests and activities, stereotyped, restricted and repetitive. These qualitative anomalies make up a global characteristic of human being functioning, at all times.^1^

Pediatric autism is considered a global development disorder, in which there is an abnormal and/or compromised development, which manifests itself before the child is three years old, and such disorder is more frequent in boys then girls (3-4:1). Among the Global Development Disorders we also have the Asperger syndrome, a disorder of uncertain nosological validity, characterized by a qualitative alteration in reciprocal social interactions, similar to what is seen in autism, with a restricted set of interests and activities, stereotyped and repetitive. It is different from autism itself because it is not coupled to retardation, language or cognitive development defficiency.^1^

It is known that children with Autism and Asperger Syndrome can have perception, attention and memory disorders, and often times the presence of peripheral and/or central hearing loss is suspected.

Evoked auditory potentials can be classified into early onset, mid-onset and late onset.^2^ The two main reasons to use these tests are: to establish the acoustic signal detection threshold and to infer on the functional and structural integrity of the auditory pathway neural components.^3^

Brainstem Auditory Evoked Potential (BEAP), considered the most commonly used evoked auditory potential in clinical practice, is an objective test that assesses the hearing pathway integrity from the auditory nerve to the brain stem, which is very much used to evaluate newborns and children with neurologic and psychiatric disorders, who are difficult to assess by means of routine audiologic procedures.^4^

The Mid-Latency Evoked Auditory Potential (MLEAP) is a series of waves that happen between 10 and 80 milliseconds (ms) after the acoustic stimulus onset. The multiple MLEAP generators include the thalamus-cortical auditory pathway, the mesencephalic reticular formation, and the inferior colliculus, being clinically used to establish the auditory electrophysiological threshold in the low frequency range, in assessing the auditory pathway functioning and possible location of lesions in this path, also helping with the diagnosis of syndromes that may compromise the wave generation system and intra-operative monitoring.^3^

Long Latency Evoked Auditory Potentials (LLEAP), also called late potentials, happen between 70 and 500ms after auditory stimulation, appearing after the MLEAP. Although the sites that generate these potentials are not well known, they are used clinically to diagnose specific development alterations, and they can indicate language alterations. The P300 is a late and endogenous potential and, attention, auditory discrimination, memory and semantic perspective seem to be associated with the generation of such potential.^5^

Thus, the goal of the present study is to describe the results obtained from hearing audiologic and electrophysiological evaluations, looking for peripheral and/or central auditory alterations in individuals with psychiatric disorders and to compare such results with those from individuals in normal development from the same age range.

## METHODS

This study was approved by the Ethics Committee of our institution, under research protocol #455/03. The evaluations were carried out after the parents/guardians signed the Informed Consent Form.

The material from the present study is based on the results of audiologic and electrophysiological evaluations of 40 individuals, aged between eight and 19 years: ten with autism (AG) - nine males and one female; ten with Asperger Syndrome (ASG) - nine males and one female; and 20 in the control group (CG) - 3 males and 17 females. Inclusion criteria for groups AG and ASG were: be within the established age range and have a medical diagnosis of Pediatric Autism with lower cognitive impairment or have a medical diagnosis of Asperger Syndrome. Inclusion criteria for the control group were: be within the established age range and have a past of normal neuropsychomotor development, no psychiatric, neurologic, speech, audiologic and auditory processing disorder.

During the interview we collected the patient’s clinical history and we inspected the external auditory canal with a Heine otoscope. Acoustic immittance measures were carried out by means of a GSI 33, Grason-Stadler device, encompassing tympanometry with the 226 Hz tone probe, and the study of the stapedial muscle acoustic reflex (ipsi and contralateral) in the frequencies of 500; 1,000; 2,000 and 4,000 Hz. In the threshold tonal audiometry, carried out in a sound-treated booth, we assessed the frequencies from 250 to 8,000 Hz through air conduction; and 500 to 4,000 Hz through bone conduction (in the frequencies with air conduction thresholds above 20 dB HL). Vocal audiometry was carried out through the Speech Recognition Threshold (SRT) and Speech Recognition Percentage Index (SRPI).

Hearing electrophysiological assessment started after the end of the audiologic evaluation, using the following procedures: BEAP, MLEAP and the P300 Cognitive Potential, carried out with the Traveler Express device from Biologic. The auditory evoked potentials were obtained with the individuals seating in a reclining chair, inside a sound-treated and electrically-treated room. The skin’s surface was cleaned with abrasive paste and the electrodes were fixed by means of an electrolytic paste and adhesive tape (MicroporeT). The acoustic stimuli were presented by a pair of supra-aural phones; model TDH-39, causing the responses. The first potential performed was the P300, followed by MLEAP and, for last, BEAP. Impedance values for the electrodes were checked before the beginning of each test, and it had to be bellow 5 kOhms. In order to obtain the P300 we used the “tone burst” stimulus at 75 dB nHL, in the frequencies of 1,000 Hz (frequent stimulus) and 1,500 Hz (rare stimulus), presented in a random fashion by the computer, with an analysis window of 512ms, high-pass filters of 30.00 Hz and low-pass filters of 1.00 Hz, and 15,000 gain, being employed a total of 300 stimuli. The electrodes were placed in the vertex (Cz), in the right and left mastoids (M2 and M1) and on the forehead (Fpz), according to the International Electrode System (IES) 10-20 international standard, being considered as active electrode, the one on the mastoid of the ear being studied; reference electrode is the one on the vertex and ground electrode is the one fixed to the forehead. Of the 300 stimuli presented, 15% to 20% were associated with the rare stimulus. Each individual was instructed to identify it, counting silently or raising the hand every time it appeared.^6^ We noticed the presence and absence of this potential, as well as its latency, when present.

For the MLEAP, the stimulus used was the click, presented in a single ear at 70 dB n HL, at a presentation speed of 10 clicks per second, and a total of 1000 stimuli were employed. The analysis window was of 9,9840ms, high pass filters of 150.00 Hz and low pass of 10.00 Hz, and a gain of 100.000. The electrodes were positioned on the right and left mastoids (M2 e M1), on the right and left temporoparietal junctions (C4 and C3) and on the forehead (Fpz), according with standard IES 10-20, being considered as active electrode the ones fixed to the mastoids, reference electrodes were the ones fixed to the temporoparietal junctions, the ground electrode was fixed to the forehead.^7^ The results were analyzed as from the Pa wave amplitude and latency, since this one is usually a higher amplitude wave and, therefore, the one most easily seen.^3^ Although the specialized literature reveals that the contralateral modalities are the ones most indicated to analyze the Pa wave^8^, this one was obtained from both modalities, ipsi and contralateral - C3/M1, C4/M2, C3/M2, C4/M1). In order to obtain the BEAP, we used the rarefact modality click stimulus, monaurally represented at 80 dB n HL, at a presentation velocity of 19 clicks per second, 0.1ms duration, and a total of 2,000 stimuli were used. The analysis windows was of 10,240ms, 3,000 Hz high pass filters and 100.000 Hz low pass filters, and a gain of 150.000. The electrodes were positioned on the forehead (Fpz) and on the right and left mastoids (M2 and M1), according with the IES 10-20 standard, being considered an active electrode fixed to the mastoid of the tested ear; the reference electrode was the one fixed to the forehead, the ground electrode was fixed to the contralateral ear of the tested one.^7^ We obtained two recordings for each side in order to check the reproducibility of the traces and, consequently, the presence of waves. We checked the absolute latencies of waves I, III and V, and interpeak intervals I-III, III-V and I-V.

The individual was considered altered when at least one of the ears, or one of the sides, had some alteration. The results from the audiologic evaluations were analyzed in a qualitative fashion, while the results from the electrophysiological evaluations were analyzed in a qualitative and quantitative fashion for all the groups. The qualitative data was analyzed by comparing the normal and altered results from each group and between the groups, in all the hearing assessments. Moreover, we compared the types of alterations seen in each group and between the groups; however, only in the electrophysiological evaluation of hearing. For the analysis of the qualitative variables we used the equality of two proportions test and the confidence interval for proportions.

We also analyzed the quantitative data by calculating the mean, median, standard deviation, lower threshold, upper threshold, minimum and maximum values of the results from each auditory evoked potential for each group. Moreover, we compared the mean values among the groups and we checked the significance levels for each comparison. In order to analyze the quantitative variables, we used the Wilcoxon tests, the Mann-Whitnney test and the confidence interval for the mean value. The significance level adopted was 0.05 (5%) and all the confidence intervals were built with 95% of statistical confidence.

## RESULTS

In relation to the audiologic evaluation results, we found a statistically significant difference between the normal and altered results for groups GC, GA and GSA, having in mind that the individuals assessed presented normal results. Thus, it was not possible to compare groups GC and GA, as well as GC and GSA.

Results from the hearing electrophysiological evaluations were characterized separately, and this part was divided in three, one for each auditory evoked potential carried out (BEAP, MLEAP and P300).

Part I - Characterizing Brainstem Evoked Auditory Potential in the control, autism and Asperger syndrome groups.

On Table 1, we find the normal and altered BEAP results for groups control, autism and Asperger syndrome.

**Table 1:** Normal and altered results in the BAEP in the groups: Control, Autism and Asperger syndrome

BAEP	Control Group		Autistic Group		Asperger syndrome group	
	N	%	N	%	N	%
Normal	20	100%	5	50%	7	70%
Altered	0	0%	5	50%	3	30%
Total	20	100%	10	100%	10	100%

p-value	<0,001*		1,000		0,074	

The alterations present in groups GA and GSA were of the low brainstem type. It was not possible to compare the types of alterations among groups GC and GA and GC and GSA, since the GC did not present altered results.

In analyzing the quantitative data, comparing waves I, III and V absolute latencies and interpeaks I-III, III-V and I-V, between the right and left ears in groups GC, GA and GSA, we did not observe statistically significant differences. Thus, in order to compare the groups, we considered the absolute latencies and interpeak values from both ears. When we compared the absolute latencies values of waves I, III and V and interpeaks I-III, III-V and I-V among groups GC and GA and GC and GSA, we observed statistically significant mean differences for waves I and III and interpeak III-V waves absolute latencies, as well as a tendency towards a statistically significant difference for interpeak interval I-III, between GC and GA. We also noticed a statistically significant difference for wave V absolute latency values, between GC and GSA.

Part II - Characterizing the results from the Middle Latency Evoked Auditory Potential in the control, autism and Asperger syndrome groups.

Following, we show the data associated with the analyses of the qualitative data from the MLEAP (Table 2).

**Table 2:** Normal and altered results in the MLEAP in the Groups: Control, Autism and Asperger syndrome.

MLEAP	Control Group		Austistics Group		Asperger S. Group	
	N	%	N	%	N	%
Normal	9	45%	7	70%	6	60%
Altered	11	55%	3	30%	4	40%
Total	20	100%	10	100%	10	100%

p-value	0,527		0,074		0,371	

When we compare GC and GA (p-value=0.196) and GC and GSA (p-value=0.439), we do not see a statistically significant differences (Table 3).

**Table 3:** Types of alterations found in the MLEAP, in groups: Control, Autism and Asperger syndrome.

MLEAP	Types of alterations						
	Ear Effect		Electrode Effect		Both		Total Number of Individuals
	N	%	N	%	N	%	
Control	1	9,1%	3	27,3%	7	63,6%	11
Autism	3	100,0%	0	0,0%	0	0,0%	3
Asperger	1	25,0%	0	0,0%	3	75,0%	4

For GC, we found a statistically significant difference when we compared the occurrence of type Both and Ear Effect alterations (p-value=0.008). For GA, we noticed a statistically significant difference when we compared the Electrode Effect and the Ear Effect alterations (p-value=0.014), and Both and Ear Effect (p-value=0.014). In Group GSA, when we compare the types of alterations, we find a statistically significant difference between the Both and Electrode Effect types (p-value=0.028). When we compared the groups in relation to the types of alterations found, we found a statistically significant difference between GC and GA for the Ear Effect type alteration (p-value=0.002), as well as a significant tendency for the type Both alteration (p-value=0.051).

As far as the quantitative data analyses are concerned, when we compare the values of the Na-Pa amplitude, for the C3/M1 and C3/M2, and C4/M1 and C4/M2 modalities, we found a statistically significant difference between Na-Pa amplitudes when we compare modalities C4/M1 and C4/M2 for GSA (Table 4) and a tendency towards a statistically significant difference for this GA comparison at the GA (p-value=0.052). In comparing Na-Pa amplitudes between modalities C3/M1 and C4/M1 and between C3/M2 and C4/M2, for each group, we noticed that there was a statistically significant difference for GA (p-value=0.047), between modalities C3/M2 and C4/M2. Comparing the Na-Pa amplitude values for each modality studied (C3/M1, C3/M2, C4/M1, C4/M2), among groups GC and GA and GC and GSA, we did not find statistically significant differences (Table 4).

**Table 4:** Na-Pa amplitude comparison between modalities C3/M1 and C3/M2, and between C4/M1 and C4/M2 of MLEAP, for groups: Control, Autism and Asperger syndrome.

MLEAP		Mean	Median	Standard Deviation	Quartile 1	Quartile 3	Size	CI	p-value
	C3/M1	1,88	1,61	1,06	1,29	1,98	21	0,45	0,244
Control	C3/M2	2,14	1,34	2,48	1,03	2,08	21	1,06	
	C4/M1	2,10	1,76	1,54	1,27	2,13	21	0,66	0,972
	C4/M2	2,74	1,57	3,35	1,00	1,99	21	1,43	
	C3/M1	1,90	2,26	0,66	1,44	2,32	10	0,41	0,575
Autism	C3/M2	1,76	1,55	0,76	1,23	2,10	10	0,47	
	C4/M1	1,92	1,96	0,73	1,42	2,39	10	0,45	0,052
	C4/M2	1,42	1,35	0,61	0,96	1,98	10	0,38	
	C3/M1	2,24	1,63	1,79	1,12	2,22	10	1,11	0,508
Asperger	C3/M2	4,30	1,74	5,70	1,09	3,50	10	3,53	
	C4/M1	2,06	1,70	0,96	1,53	2,63	10	0,60	0,017*
	C4/M2	1,47	1,45	0,63	0,98	1,87	10	0,39	

Part III - Characterizing the Cognitive Potential Results (P300) in the control, autism and Asperger Syndrome Groups.

On Table 5 we have the obtained results associated with the analyses of the qualitative data.

**Table 5:** Normal and Altered results in the P300, for groups: Control, Autism and Asperger syndrome

P300	Control		Autism		Asperger	
	N	%	N	%	N	%
Normal	17	85%	6	60%	6	60%
Altered	3	15%	4	40%	4	40%
Total	20	100%	10	100%	10	100%

p-value	<0,001*		0,371		0,371	

When we compare the results from GC and GA was well as GC and GSA, the differences found were not considered statistically significant (p-value=0.127, respectively).

On Table 6, we find the distribution of the types of alterations found in P300 for the groups studied.

**Table 6:** Types of alterations found in the P300, in groups: Control, Autism and Asperger syndrome

P300	Types of alterations						
	Latency delay		No response		Both		Total number of individuals
	N	%	N	%	N	%	
Control	3	100,0%	0	0,0%	0	0,0%	3
Autism	2	50,0%	2	50,0%	0	0,0%	4
Asperger	3	75,0%	1	25,0%	0	0,0%	4

In comparing the types of alterations within one group, we noticed the statistically significant difference between the lack of response alterations and latency delay (p-value=0.014), as well as Both, latency delay (p-value=0.014) in GC, and between Both and latency delay in GSA (p-value=0.028). Such differences were not observed for GA. We did not observe statistically significant differences when we compared GC and GA and GC and GSA in each alteration.

In comparing the P300 latency values between right and left ears, there was no statistically significant difference in the groups studied. By comparing the P300 wave latency values between GC and GA and GC and GSA, we noticed a statistically significant difference between GC and GSA.

## DISCUSSION

In the present study, we did not find alterations in the qualitative results from the audiologic evaluations in GC, GA and GSA, corroborating the findings from Rosenblum et al 9 On the other hand, Taylor et al.^10^ used the BEAP and observed a high incidence of hearing loss in the autistic population (30% had moderate hearing loss and 10% had severe to profound hearing loss). The results found in the present study also do not agree with the ones found by Rosenhall et al.^11^. According to the authors, 7.9% of the children have a mild to moderate hearing loss, 1.6% had unilateral hearing loss and 3.5% had profound hearing loss or complete deafness, representing a prevalence which is considerably above the one found in the general population. The authors stressed the importance of the audiologic evaluation in autistic individuals, having seen that individuals with profound hearing loss must be referred to hearing rehabilitation and the individuals with mild to moderate hearing level must be followed up because of the level of hearing deterioration.

In analyzing the BEAP qualitative results, we noticed that GA and GSA presented altered results, while GC had only normal results. Moreover, we observed a statistically significant different when we compare GC and GA and GC and GSA.

In the present investigation, 50% of the autistics and 30% of the individuals with Asperger Syndrome evaluated had alterations in this potential, findings which were similar to the ones obtained in other studies^12^, ^13^.

As to the types of alterations found, we know that the only type of alteration seen in GA and GSA was the Low Brainstem type (LBS), and there was a statistically significant difference between the LBS and the other alterations (High Brainstem and both).

Comparing the mean values from absolute latencies and interpeaks among GC and GA and GC and GSA we notice the existence of statistically significant mean differences for the I and III wave latency values and those for III-V interpeak when we compared GC and GA. Thus, these results indicated that autistic individuals had a delay in their nervous pulse conduction, especially in regions of the low brainstem. Such results agree partially with those found by Rosenblum et al.^9^, which described an increase in latency times from waves III and IV in autistic individuals, as well as the ones presented by Taylor et al.^10^, who found an increase in interpeaks I-III and I-V in autistic individuals. Studies^12^, ^14^ have shown, respectively, an increase in the mean latency value for wave V and an increase in interpeak I-III, in the group of autistic individuals, when compared to the control group. We stress that there is a major prevalence of studies that point towards a brainstem dysfunction in individuals with autism.

The results from the present investigation corroborate those found by Coutinho et al.^15^, which results indicated normal absolute values for waves I, III and V and interpeaks I-III, III-V and I-V in two autistic individuals.

For GSA, we noticed a statistically significant difference for the latency values of waves III and V, when compared to GC, in agreement with the results presented by Gillberg et al.^13^. There are only very few studies which aimed at investigating the results obtained from evoked auditory potentials in individuals with Asperger syndrome. Although such syndrome is considered by many authors as being part of the autistic spectrum, according with CID-101, it is different from autism, essentially for the fact that it is not coupled to mental retardation, speech deficiency or cognitive development impairment.

As far as the MLEAP is concerned, it is important to stress the large number of altered results found in the GC, and there was no statistically significant difference between normal and altered results for this group. According to many authors^16^, ^17^, the MLEAP is a useful instrument in the assessment of the auditory processing (AP). Considering that, for the present study, the auditory processing behavioral assessment was not performed, we can hypothesize that GC individuals can have PA alterations, which justify the alterations found at the MLEAP.

We noticed a tendency towards significance when we compared the normal and altered results found in GA. When we compared GC and GA (p-value=0.196), and GC and GSA (p-value=0.439), we did not see any statistically significant difference. It is known that autistic individuals have the following behavioral characteristics: speech disorders, language impairments, perception and development disorders, social relationship problems^18^, these disorders can be associated with auditory processing alterations. Thus, the alterations found in the MLEAP can be a reflex of auditory processing difficulties in cortical and subcortical regions.

In GA, the most commonly found alteration was the Ear Effect type (100%). When GC and GA were compared, we observed a statistically significant difference for the Ear Effect type of alteration (p-value=0.002). In the analysis of the MLEAP quantitative data, comparing the amplitude mean values among GC and GA and GC and GSA, we did not find statistically significant differences. In a study previously performed^19^, statistically significant differences were not found between the autistic and control groups for the latency and amplitude values of the Pa component (Pa-Nb amplitude), notwithstanding, we found abnormalities in the P1 Components (P1-Nb amplitude) in autistic individuals, suggesting that the reticular ascending activation system and/or its post-synaptic thalamic target may be dysfunctional in this population. The Both type of alteration was the one most commonly found among the GSA (75%). In the specialized literature we did not find studies that have investigated the MLEAP in this population.

As far as the P300 is concerned, all the groups assessed had altered results, and only for GC we noticed a statistically significant difference between normal and altered results, with a higher rate of normal results (85%). In both GA and GSA, we noticed 40% of altered results. Comparing GC and GA and GC and GSA we did not see statistically significant differences. Researchers^20^ have reported the presence of P300 alterations in autistic subjects when compared to the responses from control subjects. According to such researchers, these results match the idea that there are hearing alterations in the autistic patient who can, in some cases, involve low levels of neural transmission which can manifest as abnormalities involving high processing aspects, associated with the recording and storage of auditory information. Moreover, they suggest that severe speech alterations in children can be secondary to basic deficits in auditory processing.

Critchley et al.^21^ used functional MRI and noticed that autistic individuals and those with Asperger syndrome have significantly different activities in the cerebellar, mesolimbic and temporal cortex regions, concluding that such differences seem to be associated with the person’s neural development. Considering such biological differences and the temporal cortex participation in auditory information processing, one can infer about a possible correlation between the abnormal brain activities presented by these individuals and the occurrence of altered P300 results.

Gage et al.^22^ obtained empirical evidence that the maturation of the cortical auditory system in autistic children can follow a different pathway when compared to normal children, especially in the right hemisphere, and concluded that such results suggest that speech alteration, as well as the atypical sensitivity to sound that autistic individuals have, can be associated with abnormalities in the cortical auditory processing.

Jansson-Verkasalo et al.^23^, in a study with evoked auditory potentials indicated that children with Asperger syndrome have alterations in the coding of the sound transient characteristic, as well as the sound discrimination and concluded that the sensorial auditory processing is deficient in these children and that such deficits can be associated to the perception problems presented by such children with this syndrome.

Alterations like the P300 wave latency delay were the most frequently seen in the GSA (75%) and corresponded to 50% of the alterations presented by autistic individuals. Such findings show that these individuals had alterations which reflect their attention difficulties and sometimes cognitive ones, having seen that the P300 wave latency delay indicates the existence of a possible deficit in the cognitive processing^24^. Authors^5^ stressed the P300 importance for the study of cognitive and attention functions, since the auditory discrimination process, memory, semantic perspective and attention are directly associated with the generation of this potential.

When we compared the P300 latency values from GC and GA, there were no statistically significant difference. This finding corroborated those presented by Niwa et al.^25^, Erwin et al.^26^ and Ferri et al.^27^.

Comparing the P300 wave latency values from GC and GSA, statistically significant differences were found. Thus, the present study has shown discrepant results when the qualitative and quantitative data are compared. This discrepancy is probably associated to the method used to make the different analyses. Having in mind the large range of values within which the P300 wave latency is considered normal, when one calculates the mean value for wave latency for each group, one has to consider a very diversified summation of values, because very different numeric values can get the same classification, such as, for example, normal. Thus, the mean values among the groups showed a significant difference, because it is very likely that many individuals had borderline latency values. Now, for the qualitative data analyses, the variables were only two (normal and altered).

Kujala et al.^28^, when performing long latency evoked potentials with similar paradigms to the P300 in individuals with Asperger syndrome noticed a large occurrence of altered results, and the most common alterations were amplitude reduction and latency increase.

It is important to stress that, although some comparisons made in this study did not yield statistically significant differences between the control and study groups (autism or Asperger syndrome), a great part of the specialized literature investigated pointed towards the existence of alterations in the central auditory nervous system, especially at cortical and subcortical levels in the populations studied. Moreover, it is necessary to carry out more studies in order to investigate the central auditory function in these individuals, especially in relation to the Asperger syndrome, which has a very restricted literature, in relation to auditory evoked potentials.

The utilization and association of different assessment methods, objective and subjective ones, are important in order to identify alterations in the central and peripheral auditory systems, and to characterize the hearing function in special populations, especially when we consider the existence of language alterations, because hearing alterations can compromise language acquisition and development, as well as the entire rehabilitation process.

## CONCLUSIONS

Having the aforementioned results, we can conclude that individuals with autism and Asperger syndrome: did not have alterations in their audiologic evaluations, thus suggesting peripheral hearing pathway integrity and normal auditory thresholds; had alterations in the BEAP, suggesting auditory pathway involvement in the low brainstem, especially related to alterations in the synchronicity regarding the generation and transmission of neuroelectrical pulses throughout the auditory pathway in the brainstem; had large frequency of MLEAP alterations, although we did not see any statistically significant difference when compared to the control group, having seen that such group also presented an important occurrence of alterations in this potential; had large occurrence of P300 alterations, although statistically significant differences were not seen when they were compared to the control group during the analyses of the qualitative data, suggesting auditory pathway involvement in cortical regions and auditory processing deficit regarding the hearing information.
